# Highly Proliferative Immortalized Human Dental Pulp Cells Retain the Odontogenic Phenotype when Combined with a Beta-Tricalcium Phosphate Scaffold and BMP2

**DOI:** 10.1155/2020/4534128

**Published:** 2020-02-15

**Authors:** Xiangfen Li, Liu Wang, Qin Su, Ling Ye, Xuedong Zhou, Dongzhe Song, Dingming Huang

**Affiliations:** ^1^State Key Laboratory of Oral Diseases & National Clinical Research Center for Oral Diseases, West China Hospital of Stomatology, Sichuan University, Chengdu 610041, China; ^2^Department of Endodontics, West China Hospital of Stomatology, Sichuan University, Chengdu 610041, China

## Abstract

Human dental pulp cells (HDPCs) play a vital role in dentin formation and reparative dentinogenesis, which indicated their potential application in regenerative medicine. However, HDPCs, which can only be obtained from scarce human pulp tissues, also have a limited lifespan *in vitro*, and stem cells usually lose their original characteristics over a large number of passages. To overcome these challenges, we successfully immortalized human dental pulp cells using the piggyBac system which was employed to efficiently overexpress the SV40 T-Ag, and we then comprehensively described the cell biological behavior. The immortalized human dental pulp cells (iHDPCs) acquired long-term proliferative activity and expressed most HDPC markers. The iHDPCs maintained multiple differentiation potential and could be induced to differentiate into chondrogenic, osteogenic, and adipogenic cells *in vitro*. We also proved that the iHDPCs gained a stronger ability to migrate than the primary cells, while apoptosis was inhibited. Furthermore, highly proliferative iHDPCs displayed no oncogenicity when subcutaneously implanted into athymic nude mice. Finally, iHDPCs exhibited odontogenic differentiation ability and secreted dentin sialophosphoprotein (DSPP) when combined with a beta-tricalcium phosphate scaffold and bone morphogenetic protein-2 (BMP2) *in vivo*. Conclusively, the established iHDPCs are a valuable resource for mechanistic study of dental pulp cell differentiation and dental pulp injury repair, as well as for applications in tooth regeneration.

## 1. Introduction

The stem cells in teeth have prospects for broad application in regenerative medicine. Human dental pulp cells (HDPCs), comprising a cell mixture of undifferentiated dental pulp stem cells, fibroblasts, dendritic cells, and macrophagocytes, are endowed with multiple differentiation potential. Among the multitudinous tooth-related stem cells, HDPCs have great potential for both dentin formation and regeneration [1]. Previous studies have employed multiple scaffolds for tooth and bone regeneration [1–5]. When HDPCs are recombined with biological scaffolds and implanted into a root canal, they can generate dentin pulp-like tissues *in vivo* [1, 2]. Indeed, osteogenic differentiation of HDPCs will be induced by bECM hydrogel without the addition of growth factors [4]. Meanwhile, HDPCs are regarded as the ideal cell source for many areas of regenerative medicine beyond the tooth due to the fact they can be easily isolated from discarded teeth. However, HDPCs have a limited lifespan *in vitro* and stem cells usually lose their original characteristics after many passages [1]. Consequently, it is necessary to seek efficient methods to harvest enough HDPCs from a limited supply of pulp tissue and to expand the cells sufficiently to provide an adequate number of cells *in vitro* to cater to these urgent needs.

Immortalization of various cells without losing their stemness has been adopted to solve these problems thanks to developments in technology. Although some attempts have been made to obtain human dental pulp cells and culture them *in vitro*, few *in vivo* applications of immortalized human dental pulp cells have been reported, and there is also a lack of comprehensive descriptions of their biological behavior [6–8]. Immortalization of cells can be achieved by means of oncogene overexpression and tumor suppressor gene inhibition [9]. The SV40 T-Ag has been recognized as the most widely used gene for immortalization [10–12]. Unfortunately, due to the low viral titers of a retrovirus when a long gene fragment is transduced, the primary cells have a low immortalization efficiency when a retroviral vector is employed to overexpress the SV40 T-Ag [11, 13–17]. Thus, the major challenge to immortalization of cells is the search for a simple, efficient, and convenient method to transfer the immortalizing genes into cells [18].

The piggyBac (PB) system is composed of mutant baculovirus strains derived from the cabbage looper moth *Trichoplusia ni* [19]. PB transposition, endowed with host factor-independent characteristics, is regarded as the most popular nonviral gene delivery tool [20]. The SV40 T-Ag gene located between two flippase recognition target (FRT) sites and delivered by the vector pMPH86 has contributed to the immortalization of various human and mouse cells [16].

The aim of this research was to immortalize human dental pulp cells efficiently and safely using a PB-based gene delivery system. In addition, experiments were carried out to test the characteristics of immortalized human dental pulp cells (iHDPCs) and explore the feasibility of applying these iHDPCs in tooth regeneration. In this study, we elucidated the biological behavior changes of iHDPCs which hinted at some superior characteristics in pulp regeneration. Meanwhile, cells combined with beta-tricalcium phosphate (*β*-TCP) were transplanted subcutaneously to test the odontogenic feasibility of iHDPCs *in vivo*. Our results demonstrated that iHDPCs, which have superior biological performance, might be a valuable resource to explore the mechanism of dental pulp cell differentiation and dental pulp injury repair, as well as for application in the future study of pulp regeneration.

## 2. Materials and Methods

### 2.1. Ethics Statement

Human pulp tissues were collected from patients at West China Hospital of Stomatology under approved guidelines. The research was approved by both the Ethical Committees of West China School of Stomatology, Sichuan University, and the State Key Laboratory of Oral Diseases.

### 2.2. Establishment of Reversibly Immortalized HDPCs

Dental pulp was collected from donors (aged 18–22 y), and human primary dental pulp cells were isolated and cultured in Dulbecco's modified Eagle's medium (DMEM) (HyClone, Logan, UT, USA) supplemented with 10% fetal bovine serum and 100 U/mL penicillin and 100 U/mL streptomycin [21]. To establish the iHDPCs, early passages of human dental pulp cells (passage 3) were transfected with the PB vector pMPH86 and the PB transposase expressing the adenoviral vector AdpBase. Then, hygromycin B (4 mg/mL, Gibco/Life Technologies, Carlsbad, CA, USA) was added for 3 days to select stably immortalized HDPCs. To establish deimmortalized human dental pulp cells (dHDPCs), iHDPCs were infected with Ad-FLP which effectively recognized the FLP site and removed the SV40 T-Ag. Cell morphology and growth features were observed under an inverted microscope. Total RNA was extracted, and real-time reverse transcription polymerase chain reaction (qRT-PCR) and agarose gel electrophoresis were performed to test the SV40 T-Ag gene expression level.

### 2.3. Surface Antigen Expression Assay

IHDPCs were cultured in 60 mm dishes until 70% confluence. Cell surface antigens were detected by immunofluorescence. Primary antibodies against CD90, CD105, CD34, CD45 (all at 1 : 100, Abcam, Cambridge, MA, USA), and CD73 (1 : 100, BioLegend, San Diego, CA, USA), a goat anti-mouse lgG secondary antibody fluorescent labeled with Alexa Fluor 555 (1 : 1000, Invitrogen, Carlsbad, CA, USA), or a goat anti-rabbit lgG secondary antibody fluorescent labeled with Alexa Fluor 488 (1 : 1000, Invitrogen) was used. Flow cytometry of HDPCs and iHDPCs was performed as described previously [22].

### 2.4. Cell Counting Kit-8 (CCK8) Proliferation Assay

Three thousand cells per well were seeded into 96-well plates. Cells were continuously observed for 5 days to determine cell viability, and 10 *μ*L of CCK8 solution (Dojindo Laboratories, Kumamoto, Japan) was added into the medium daily followed by 1.5 h incubation. The absorbance of the medium at 450 nm was measured with a microplate reader (BioTek, Winooski, VT, USA).

### 2.5. Colony Formation Assay

HDPCs, iHDPCs, dHDPCs, and iHDPCs infected with Ad-GFP were seeded into 6-well plates at a density of three hundred cells per well. Cells were fixed in 4% paraformaldehyde for 15 min after culture in growth medium for 3 weeks. Then, crystal violet staining (Beyotime Institute of Biotechnology, Jiangsu, China) was performed and recorded using a bright-field microscope.

### 2.6. Crystal Violet Assay

Subconfluent HDPCs, iHDPCs, dHDPCs, and iHDPCs infected with Ad-GFP were seeded into 6-well plates. At the indicated time points, all of the cells were fixed and subjected to crystal violet staining. The stained cells were evaluated by quantitative measurement after dissolving the stain in 10% acetic acid for 20 min with agitation and then measuring the absorbance value at 570 nm.

### 2.7. Flow Cytometric Analysis

Before analysis of cell cycle distribution, cells were fixed in ice-cold 70% ethanol for 2 hours. After washing with PBS, cells were incubated with RNase (KeyGen Biotech Co. Ltd., Nanjing, China) for 30 min at 37°C followed by incubation with propidium iodide (PI) (KeyGen Biotech) for 30 min at 4°C. The results were examined on a Guava easyCyte HT flow cytometer (Merck-Millipore, Darmstadt, Germany) and analyzed with InCyte 2.7 software (Millipore). For analysis of apoptosis, cells were stained with Annexin V and 7-AAD (both from KeyGen Biotech) according to the manufacturer's instructions. Apoptotic fractions were analyzed using a FACScan cytometer (Becton-Dickinson, Franklin Lakes, NJ, USA). In our studies, the early apoptotic cells (Q4: Annexin V+/7-AAD-staining) and the late apoptotic cells (Q2: Annexin V+/7-AAD+staining) were considered to be undergoing apoptosis, and the numbers of these apoptotic cells as a proportion of total cells were analyzed.

### 2.8. Scratch Wound Healing Assay

Cells were cultured in 60 mm dishes at a density of 1 × 10^6^ cells per well until 90% confluence. A 10 mL plastic pipette was used to create a wound across the diameter of the plate. After washing with medium to remove debris, cell migration was observed under a microscope after a 24 h interval.

### 2.9. *In Vitro* Multidifferentiation of iHDPCs

Cells were cultured in twelve-well plates at a density of 1 × 10^5^ cells per well. To induce adipogenic differentiation, the medium was replaced with basal *α*-modification of Eagle's medium plus 100 nM dexamethasone, 50 *μ*g/mL ascorbic acid 3-phosphate, and 50 *μ*g/mL indomethacin (Sigma-Aldrich, St. Louis, MO, USA) for 2 weeks. The differentiated cells were fixed with 4% polyoxymethylene for 15 min before staining with 0.3% Oil Red O (Sigma-Aldrich) solution to evaluate adipogenesis. To induce chondrogenic differentiation, the cells were incubated in the presence of chondrogenic differentiation medium (Lonza, Basel, Switzerland) with recombinant transforming growth factor beta-3 protein (R&D Systems, Minneapolis, MN, USA) for 2 weeks. The induced cells were fixed with 4% polyoxymethylene for 15 min, followed by staining with 1% Alcian blue (Sigma-Aldrich). To induce osteogenic differentiation, the cells were incubated in the presence of 10 nM dexamethasone, 50 mg/mL ascorbic acid 2-phosphate, and 10 mM *β*-glycerophosphate (all from Sigma-Aldrich) for 5 days. Then, the differentiated cells were fixed with 4% polyoxymethylene for 15 min, followed by alkaline phosphatase staining (Beyotime) to assess mineral deposition according to the manufacturer's instructions.

### 2.10. Quantitative Real-Time Polymerase Chain Reaction (qRT-PCR)

To detect multipotential differentiation of HDPCs, iHDPCs, and dHDPCs, total RNA was extracted from cells using the RNeasy mini kit (Qiagen, Valencia, CA, USA) according to the manufacturer's protocol. The isolated RNA was reverse transcribed using the PrimeScript RT Reagent Kit (Takara). The complementary DNA samples were used as templates in SYBR Premix Ex Taq (Takara) PCR reactions. Glyceraldehyde 3-phosphate dehydrogenase (GAPDH) was used as an internal control.

### 2.11. Subcutaneous Tumor Formation in Nude Mice

Seven-week-old nude mice (*n* = 5) were inoculated subcutaneously with cells in 0.20 mL of PBS per mouse at the level of the scapulae. SCC-4, an established human-origin head and neck squamous cell carcinoma tumor cell line, was used as the positive control. The nude mice were observed up to 3 weeks, when there was obvious tumor formation in the positive control group. After imaging, tissues near the injection sites were collected and examined by hematoxylin and eosin (H&E) (Solarbio, Beijing, China) staining.

### 2.12. Subcutaneous Transplantation of IHDPCs

The *β*-TCP blocks were purchased from the Biological Materials Manufacturing Core, Sichuan University. Cells were infected with Ad-BMP2 in advance. The composites of *β*-TCP blocks and cells were prepared as previously described [23] and implanted into the left and right subcutaneous dorsal pockets of six-week old BALB/c immunodeficient nude mice (*n* = 5). One month after implantation, the composites were harvested and fixed with 4% formalin, followed by decalcification with 10% ethylenediaminetetraacetic acid (EDTA) for one week.

### 2.13. H&E Staining and Masson's Trichrome Staining

The tissues were embedded in paraffin. Serial sections of the embedded specimens were stained with H&E and Masson's trichrome stains (both from Solarbio) according to the manufacturer's protocol.

Images were obtained by a Nikon Eclipse 300 fluorescence microscope (Compix Media Inc., Irvine, CA, USA).

### 2.14. Immunofluorescence Staining

Paraffin sections were incubated overnight with the primary antibody anti-dentin sialophosphoprotein (DSPP) (1 : 20; Santa Cruz, CA, USA) at 4°C. This procedure was followed by secondary antibody fluorescent labeling with Alexa Fluor 555 (1 : 1000, Invitrogen, Carlsbad, CA, USA) for 60 minutes at room temperature. The cell nuclei were also labeled with diamidino-phenylindole (DAPI; Invitrogen). Images were obtained using a Nikon Eclipse 300 fluorescence microscope (Compix Media Inc.).

### 2.15. Statistical Analysis

Experimental data are presented as means ± SD. Significance was determined by the one-way analysis of variance test. Each assay condition was repeated in triplicate for all quantitative assays. A value of *P* < 0.05 was considered statistically significant.

## 3. Results

### 3.1. HDPCs Can Be Effectively Immortalized by the PiggyBac Transposon System

**Figure 1:**
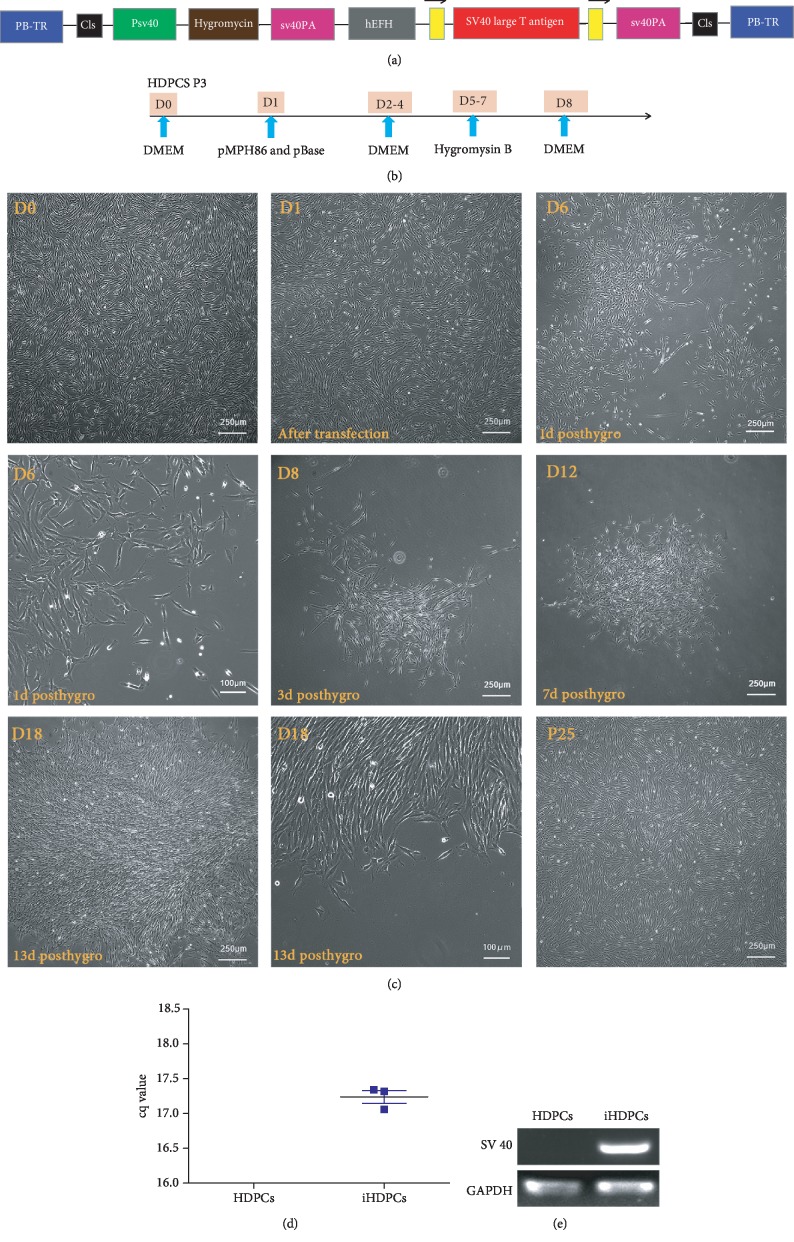
Immortalization of human dental pulp cells using the piggyBac transposon system. (a) Schematic diagram of pMPH86. The SV40 T-Ag gene is located between two flippase recognition target (FRT) sites. (b) Schematic of the experimental approach. (c) IHDPC establishment. The adenoviral vectors AdpBase and pMPH86 transfected the primary human dental pulp cells, and cells were selected in medium with hygromycin for 3 days. Surviving cells were observed at day 3, day 7, and day 13 postselection. The iHDPCs passaged consecutively for 25 passages (p25) (d and e). The mRNA expression of SV40T-Ag was upregulated after immortalization.

HDPCs were transduced with the piggyBac vectors pMPH86 (Figure 1(a)) and AdpBase and incubated in DMEM supplemented with normal serum for 3 days; then, hygromycin B was added for the next 3 days. On day 8, the positive cells were transferred to DMEM for further expansion and maintained in this medium thereafter. A schematic of the protocol is shown in Figure 1(b). After hygromycin selection, the surviving immortalized human dental pulp cells maintained a high proliferation rate and were passaged consecutively for more than 70 generations over 210 days, well beyond the Hayflick limit without any change in their fibroblast-like morphology (Figure 1(c)). Cells infected with pMPH86 exhibited a higher integration rate of SV40 T-Ag (Figures 1(d) and 1(e)).

### 3.2. IHDPCs Have a Greater Expansion Capacity Than HDPCs

**Figure 2:**
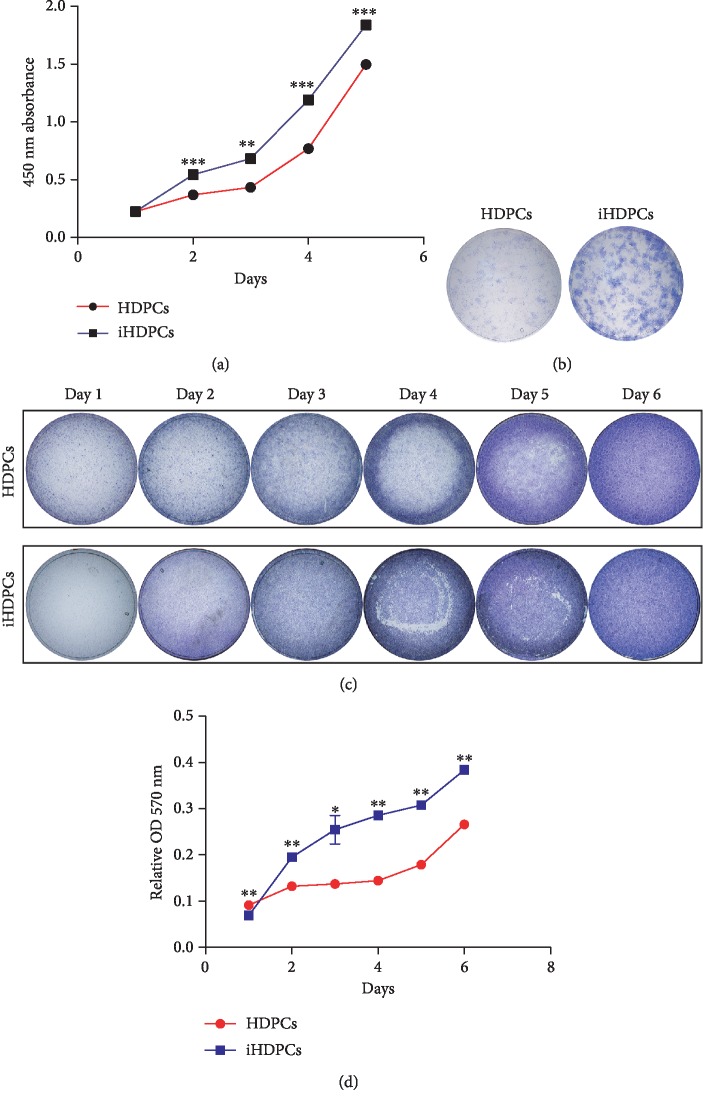
IHDPCs acquired strong self-renewal and proliferation ability. (a) Cell proliferation was observed by the CCK8 assay (^∗∗^*P* < 0.01, ^∗∗∗^*P* < 0.001). (b) Colony formation assay using crystal violet staining. (c) Cell proliferation assessed by the crystal violet staining assay. (d) The stain extracted from the cells was dissolved for quantitative determination at A570 nm (^∗^*P* < 0.05, ^∗∗^*P* < 0.01).

The CCK8 cell proliferation assay suggested that iHDPCs had stronger proliferation ability than HDPCs, especially within the first 4 days (Figure 2(a)) which was also indicated by the colony formation assay (Figure 2(b)) and the crystal violet staining assay (Figure 2(c)). Quantitative assessment supported the staining results, confirming that iHDPCs had significantly higher cell proliferation activity at day 2 than the HDPCs (Figure 2(d)).

### 3.3. Biological Behavior Changes of IHDPCs

**Figure 3:**
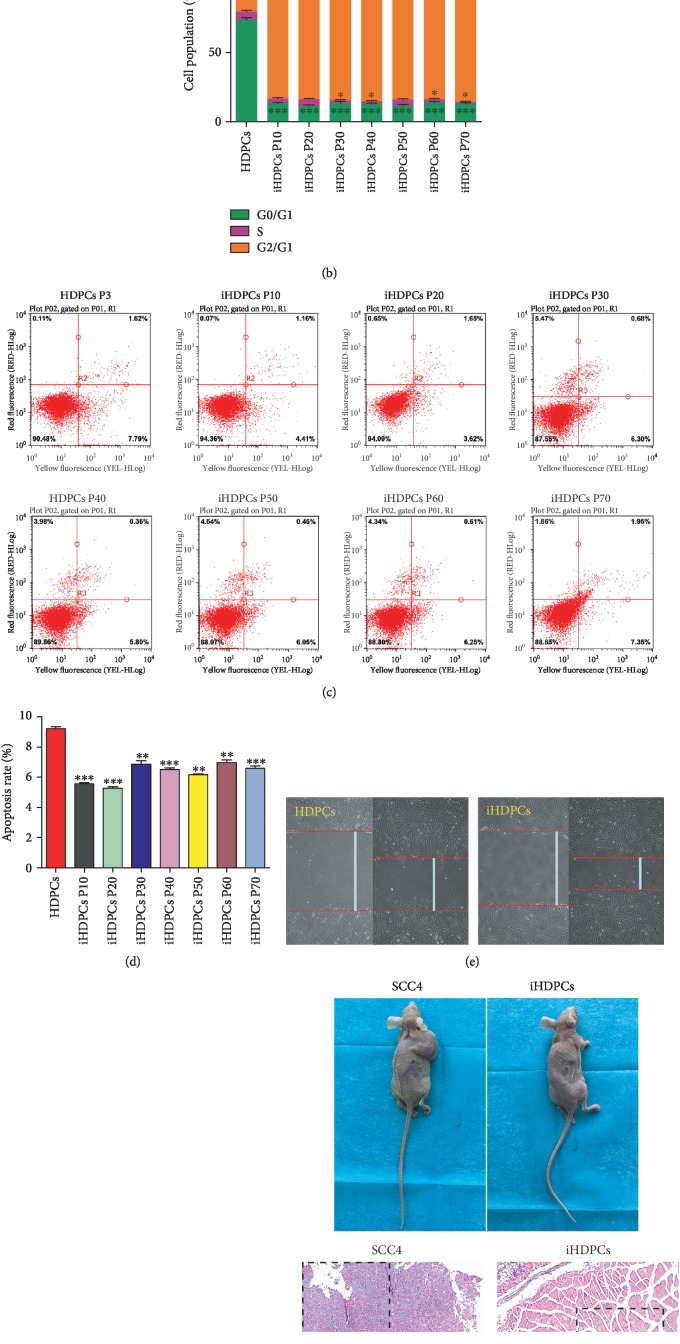
Biological behavior changes of iHDPCs. (a) Flow cytometric analysis showed the cell cycle distribution of HDPCs and iHDPCs. (b) Cell cycle analysis by flow cytometry showed increased G2-M phase fractions in the iHDPCs while the cell cycle distribution of iHDPCs was not affected by passaging (^∗∗∗^*P* < 0.001). (c) Flow cytometric analysis showed the levels of apoptosis in HDPCs and iHDPCs. (d) The percentage of apoptotic cells among iHDPCs was significantly lower than that among HDPCs and was not affected by passaging (^∗∗^*P* < 0.01, ^∗∗∗^*P* < 0.001). (e and f) The migration rates of HDPCs and iHDPCs were quantified using the wound healing assay (^∗^*P* < 0.05). (g) The nude mouse tumorigenicity assay was used to test the oncogenicity of iHDPCs, with SCC-4 used as the positive control.

IHDPCs ranging from passage 10 to passage 70 were collected for cell cycle analysis by flow cytometry. We observed higher G2-M phase fractions in the iHDPCs, and the cell cycle distribution of iHDPCs was not affected by passaging (Figures 3(a) and 3(b)). Meanwhile, the percentage of apoptotic cells among iHDPCs was significantly lower than that among HDPCs and was also not affected by passaging (Figures 3(c) and 3(d)). Furthermore, compared with HDPCs, iHDPCs had superior migration ability which was quantified by the wound healing assay (Figures 3(e) and 3(f)). The nude mouse tumorigenicity assay was used to test the oncogenicity of iHDPCs, taking the human head and neck squamous cell carcinoma cell line SCC-4 as the positive control. There were no abnormal mitoses or tumor cell formation in the iHDPC group compared with the SCC-4 group (Figure 3(g)). Thus, we concluded that iHDPCs did not have tumorigenic properties. Our data suggested that the biological behavior changes of iHDPCs might contribute much to their application in pulp tissue engineering.

### 3.4. The IHDPCs Express the Majority of Marrow Stromal Cell Markers

**Figure 4:**
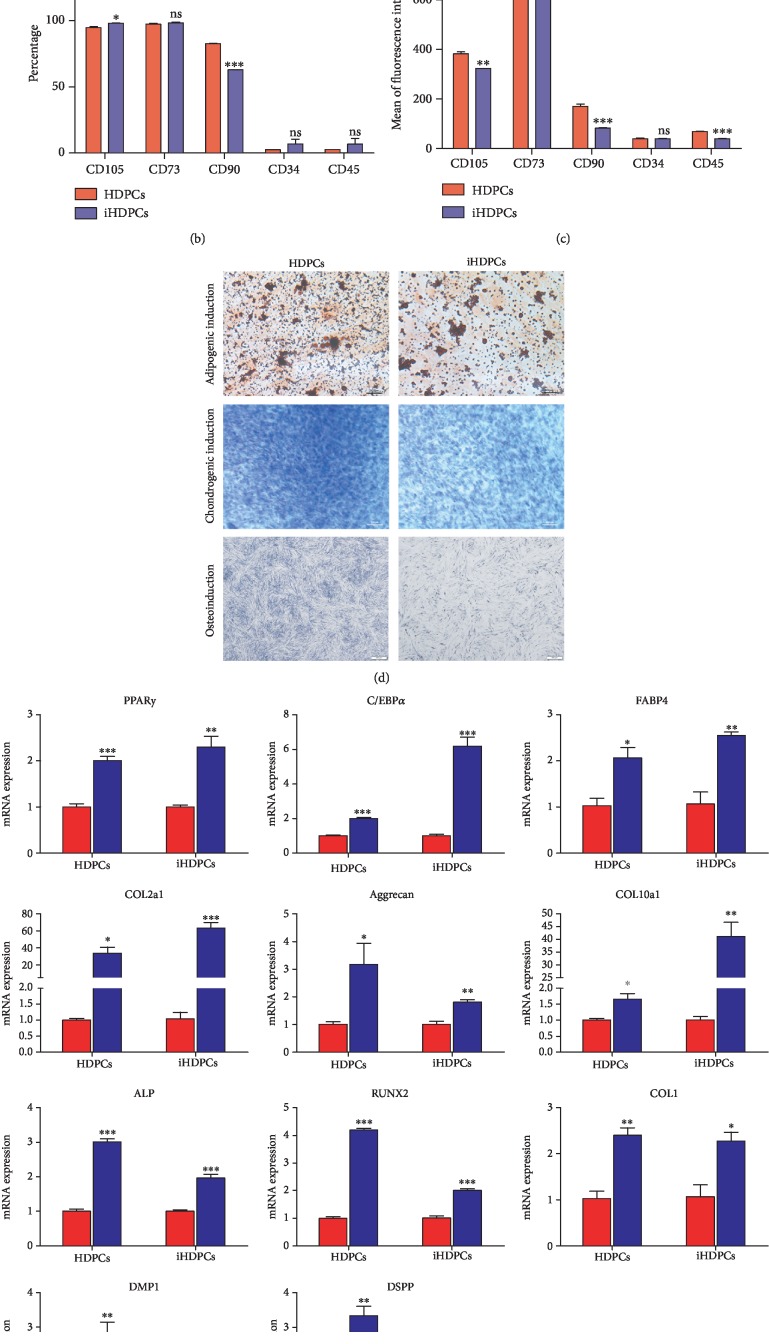
Multilineage differentiation of HDPCs and iHDPCs. (a) Flow cytometric analysis of the antigens expressed by HDPCs and iHDPCs. (b) Percentages of positive cells. (c) Mean fluorescence intensity of cells stained with the different antigens. (d) Adipogenic differentiation of HDPCs and iHDPCs determined by Oil Red O staining after culture in adipogenic medium for 14 days. Chondrogenic differentiation of HDPCs and iHDPCs determined by Alcian blue staining after culture in chondrogenic medium for 14 days. Osteogenic/odontogenic differentiation of HDPCs and iHDPCs determined by ALP staining after culture in osteogenic medium for 5 days. (e) qRT-PCR analysis of PPAR*γ*, C/EBP*α*, EABP4, collagen 2a1, aggrecan, collagen 10a1, ALP, RUNX2, COL1, DMP1, and DSPP expression (^∗^*P* < 0.05, ^∗∗^*P* < 0.01, and ^∗∗∗^*P* < 0.001).

It has been reported that consensus human dental pulp stem cell markers consist of CD37, CD90, and CD105. Negative cell surface markers include CD34 and CD45. Results of immunofluorescence staining showed that iHDPCs expressed the antigens CD37, CD90, and CD105 but did not express antigens CD34 and CD45, indicating that they were identical to primary human dental pulp cells (Figures 4(a)–4(c)).

### 3.5. The IHDPCs Are Capable of Differentiating into Adipogenic, Chondrogenic, and Osteogenic Lineages

IHDPCs were positive for Oil Red O staining, Alcian blue staining, and alkaline phosphatase (ALP) staining (Figure 4(d)). IHDPCs exhibited higher expression of peroxisome proliferator-activated receptor gamma (PPAR*γ*), CCAAT/enhancer binding protein alpha (C/EBP*α*), and fatty acid-binding protein 4 (FABP4) (genes involved in adipogenic differentiation) (Figure 4(e)) and exhibited higher expression of collagen 2a1, collagen 10a1, and aggrecan (genes involved in chondrogenic differentiation) compared with control groups (Figure 4(e)). After culture in osteogenic/odontogenic medium for 5 days, IHDPCs showed weaker osteogenic differentiation compared with HDPCs (Figure 4(d)). IHDPCs exhibited expression of ALP, runt-related transcription factor 2 (RUNX2), collagen-1 (COL1), DSPP, and dentin matrix protein-1 (DMP1) after culture in osteogenic/odontogenic medium for 5 days or 14 days (Figure 4(e)).

Taking these results together, iHDPCs were multipotent like HDPCs. However, the results demonstrated that iHDPCs were less easy to differentiate compared to HDPCs. We suspected that this may be attributable to the high proliferative ability of iHDPCs.

### 3.6. Removal of the SV40 T Antigen Mediated by FLP Recombinase Contributes to the Recovery Effect of the IHDPCs

**Figure 5:**
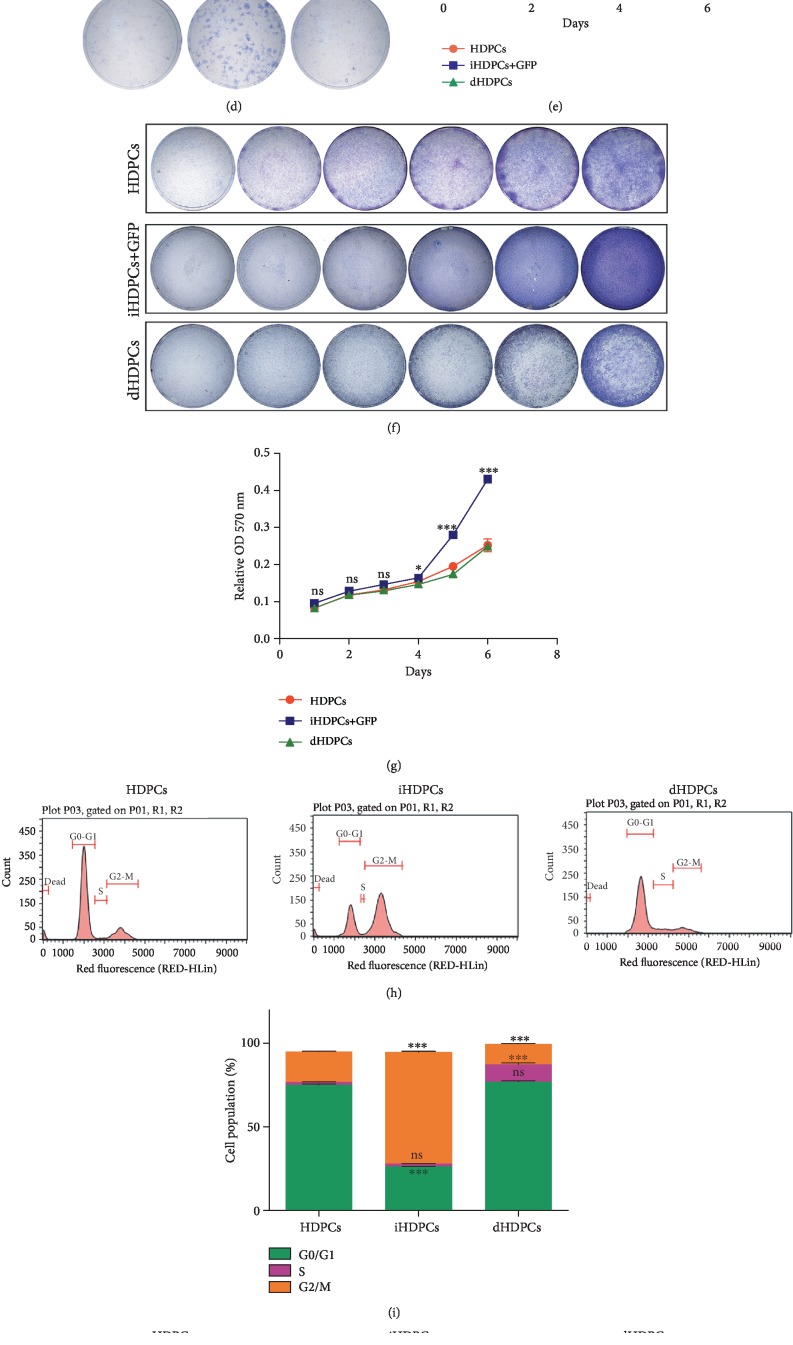
Deimmortalized human dental pulp cells. (a) IHDPCs transduced efficiently by Ad-FLP to establish deimmortalized human dental pulp cells (dHDPCs). (b) mRNA expression of SV40T-Ag was downregulated after deimmortalization (^∗∗∗^*P* < 0.001). (c) Expression of SV40T-Ag in dHDPCs. (d) The viability of dHDPCs was tested by the colony formation assay. (e) Cell proliferation assessed by the CCK8 assay (^∗^*P* < 0.05, ^∗∗^*P* < 0.01, and ^∗∗∗^*P* < 0.001). (f) Cell proliferation assessed by the crystal violet staining assay. (g) The stained cells were dissolved for quantitative determination at A570 nm (^∗^*P* < 0.05, ^∗∗^*P* < 0.01). (h and i) Flow cytometric analysis showed the cycle distribution of HDPCs, iHDPCs, and dHDPCs (^∗^*P* < 0.05, ^∗∗^*P* < 0.01, and ^∗∗∗^*P* < 0.001). (j and k) Flow cytometric analysis showed the apoptosis of HDPCs and iHDPCs (^∗∗∗^*P* < 0.001). (l) The nude mouse tumorigenicity assay was used to test the oncogenicity of dHDPCs in comparison with SCC-4 cells as the positive control. There were no developed tumors in the dHDPC group after three weeks. In contrast, there were many abnormal mitotic images in the SCC-4 group (arrows).

IHDPCs were efficiently infected with Ad-GFP or Ad-FLP (Figure 5(a)). The SV40 T antigen was efficiently removed from Ad-FLP-infected iHDPCs, compared with the Ad-GFP group (Figures 5(b) and 5(c)). The proliferative activity of the dHDPCs was reduced, as assessed by the colony formation assay, CCK8 cell proliferation assay, and crystal violet staining (Figures 5(d)–5(g)). Flow cytometric analysis showed that the cell cycle distribution of dHDPCs was similar to that of HDPCs but not iHDPCs (Figures 5(h) and 5(i)). Meanwhile, analysis of apoptosis by flow cytometry revealed that dHDPCs maintained a lower apoptosis rate (Figures 5(j) and 5(k)). As with the iHDPCs, the nude mouse tumor transplantation experiment revealed that dHDPCs had satisfactory characteristics without tumorigenesis compared with SSC4 cells (Figure 5(l)). Collectively, these results showed that dHDPCs which retain superior characteristics can be successfully established by reversing the immortalization of iHDPCs.

### 3.7. IHDPCs Can Be Differentiated into Odontoblasts When Combined with a Beta-Tricalcium Phosphate Scaffold and the Growth Factor BMP2

**Figure 6:**
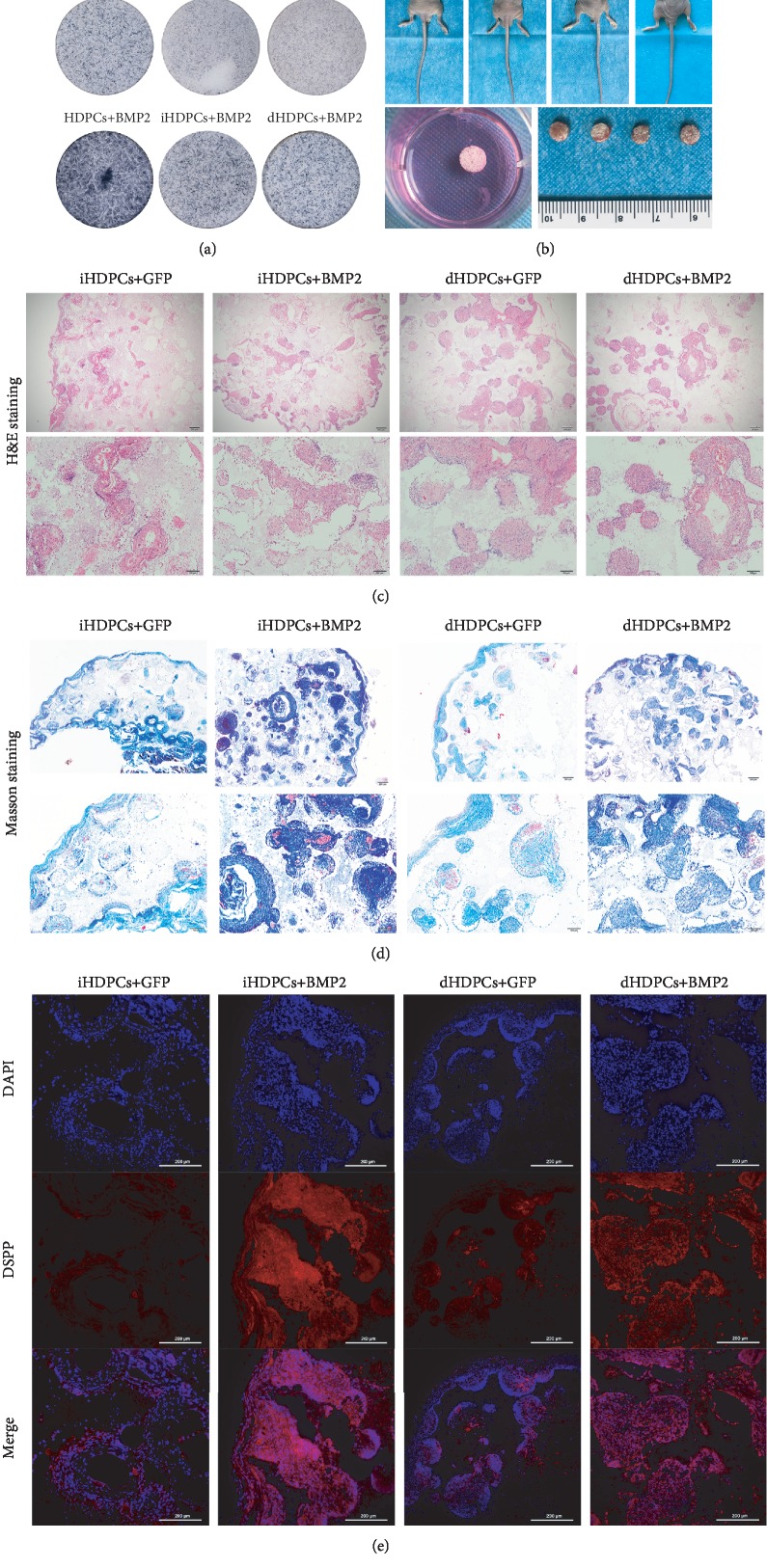
IHDPCs can differentiate into odontoblasts when combined with a beta-tricalcium phosphate scaffold and growth factor *in vivo*. (a) BMP2 promotes the differentiation and mineralization of iHDPCs and dHDPCs *in vitro*. ALP staining of iHDPCs and dHDPCs after culture in odontogenic medium for 5 days. (b) Composites of scaffolds and cells were carefully implanted into the dorsal subcutaneous region of nude mice for 4 weeks. (c) H&E staining of the composites of *β*-TCP scaffolds and iHDPCs/dHDPCs treated with BMP2 after subcutaneous implantation for 4 weeks. (d) Masson's trichrome staining of the composites. (e) The levels of DSPP in the *β*-TCP scaffold and iHDPC/dHDPC composites were analyzed by immunocytochemical staining.

As previously confirmed, the ALP activity of iHDPCs was decreased compared to that of HDPCs *in vitro* (Figure 4(d)). In order to achieve better osteogenic or odontogenic differentiation, the growth factor BMP2 was added to the iHDPCs or dHDPCs. Odontogenic differentiation of cells was assayed by ALP staining after culture in odontogenic medium for 5 days (Figure 6(a)). Finally, BMP2 effectively rescued the differentiation and mineralization of iHDPCs and dHDPCs which were impaired by immortalization *in vitro* (Figure 6(a)). To evaluate the feasibility of applying iHDPCs and dHDPCs for pulp regeneration *in vivo*, the *β*-TCP/cell composites were subcutaneously transplanted into BALB/c nude mice (Figure 6(b)). Four weeks later, we found that cells were able to grow well in the scaffold (Figure 6(c)), and more mature mineralized nodules (dark blue) were observed in the BMP2 groups compared with the GFP group (Figure 6(d)). In addition, the Ad-BMP2-infected groups showed increased expression of the odontoblast-specific marker DSPP (Figure 6(e)). Consistently, these results suggested that iHDPCs can differentiate into odontoblasts especially when used in combined applications with BMP2.

## 4. Discussion

Caries and dental trauma are high-incidence diseases leading to hard tissue injuries and pulp inflammation. Many adult stem cell sources have been applied to tissue regeneration in the oral and maxillofacial region, such as HDPCs, stem cells derived from root apical papilla (SCAP), stem cells from human exfoliated deciduous teeth (SHED), stem cells from cryopreserved periodontal ligament (PDLSCs), human periapical cyst mesenchymal stem cells (hPCy-MSCs), and oral mucosal progenitor cells [22, 24] among which HDPCs are the most common and easily obtained from extracted human teeth. HDPCs successfully regenerate a dentin pulp-like structure *in vivo* when transplanted with hydroxyapatite/tricalcium phosphate (HA/TCP) powder [25]. Based on the aforementioned properties, HDPCs not only are promising cells for tooth regeneration owing to their ability to differentiate into odontoblasts and create reparative dentin but also have attracted attention because of their ability to repair multitudinous tissues outside of the tooth [1, 26, 27]. However, isolation of adult stem cells from the human body will cause ethical discussion despite their potential applications in regeneration. Meanwhile, it is difficult to isolate enough cells from the limited human pulp tissue. Establishment of immortalized human dental pulp cells has been put forward to overcome this problem. Cell immortalization has been realized by means of spontaneous random mutagenesis of primary cells, overexpression of oncogenes, and tumor suppressor gene inhibition [18]. According to the literature, immortalized human dental pulp cells have been established with the help of a retroviral vector involved in the immortalization system [6–8]. In our research, we utilized the piggyBac transposon-mediated system (Figure 1(a)) to stably express SV40 T-Ag which has proven to be more effective than the retroviral vector-based reversible immortalization system related to the efficient immortalization of various cells including mouse embryonic fibroblasts, mouse hepatic cells, mouse cardiomyogenic cells, and mouse melanocytes [11, 14, 15, 17, 28, 29]. There are many striking features of the piggyBac transposon-mediated system [30]. Briefly, it can insert foreign fragments of DNA of up to 100 kb into the genome of human and mouse cells [28, 31] and efficiently integrate DNA elements at multiple locations [32]. Moreover, there is little evidence of mutations associated with the piggyBac transposon system owing to its infrequent integration near active genes or cancer-related genes [33]. In addition, piggyBac transposase can reversibly immortalize cells by removing the immortalization gene SV40 T-Ag with the help of FLP recombinase [18]. Thus, the piggyBac transposon system is a superior DNA transposon to deliver genes compared with other transposons [33].

The seed cells involved in tissue engineering should possess potentiality to proliferate and differentiate *in vivo*. In our research, we performed more detailed testing and description of cell characteristics involved in tooth regeneration compared with previously established immortalized HDPCs. As the results showed, iHDPCs were endowed with superior abilities of proliferation, migration, and apoptosis without tumorigenesis (Figures 2 and 3) and have been proven to express the same pattern of stem cell surface markers as primary HDPCs (Figures 4(a) and 4(b)) indicating that they contain the same amount of stem cells. Meanwhile, we were able to reversibly immortalize cells by removing the immortalization gene SV40 T-Ag. Those deimmortalized cells still retained the superior characteristics including a high rate of proliferation, low apoptosis, and odontogenic differentiation ability (Figure 5). These results suggested that iHDPCs may be an alternative to primary dental pulp cells in the field of pulp and dentin regeneration.

However, their osteogenic and odontogenic differentiation ability is not satisfactory (Figure 4(d)). There is extensive evidence in the literature that cell proliferation and differentiation are negatively correlated. Cells maintain a high proliferation rate when they are in the cell cycle, and the cell cycle exit is closely coordinated with cell differentiation [34, 35]. Therefore, we estimated that impaired odontogenic differentiation ability may be attributed to the high cell proliferative ability (Figure 2) and large G2/M-phase fraction (Figures 3(a) and 3(b)). BMP2 is one of the most commonly used BMPs and can induce odontoblast differentiation and dentin formation and the expression of DSPP and DMP1 by enhancing the activity of transcription factors [36, 37]. Thus, we opted to use BMP2 as the growth factor in our research. As expected, it achieved ideal effects in that BMP2 effectively promoted the differentiation and mineralization of iHDPCs and dHDPCs *in vitro* and *in vivo*.

Although the results support the potential use of the iHDPCs in pulp and dentin engineering, their osteogenic and odontogenic differentiation ability is not satisfactory without growth factors. So, future experiments are needed to improve cell performance to cater to superior tissue regeneration. In addition, due to the multiple factors affecting tissue homeostasis and regeneration, such as inflammatory conditions and types of growth factors, more experiments will be necessary to detect the inflammation conditions during the tissue regeneration and identify the optimum conditions for the therapeutic application of iHDPCs [38, 39].

## 5. Conclusion

We demonstrated that the established immortalized human dental pulp cells were stable, reversible, highly proliferative, and nontumorigenic cells, which should be valuable for studying the mechanisms of pulpitis, reparative dentin formation, and odontogenic differentiation of cells.

## Data Availability

The data used to support the findings of this study are included within the article.
